# The CeCORD-J study on collagenase injection versus aponeurectomy for Dupuytren's contracture compared by hand function and cost effectiveness

**DOI:** 10.1038/s41598-022-12966-z

**Published:** 2022-05-31

**Authors:** Michiro Yamamoto, Hideo Yasunaga, Ryosuke Kakinoki, Naoto Tsubokawa, Akimasa Morita, Katsumi Tanaka, Akinori Sakai, Toshikazu Kurahashi, Hitoshi Hirata, CeCORD J study Group, Hitoshi Hirata, Masahiro Tatebe, Michiro Yamamoto, Tetsuro Onishi, Katsuyuki Iwatsuki, Katsuhiro Tokutake, Hideo Yasunaga, Ryosuke Kakinoki, Kazuhiro Otani, Naoto Tsubokawa, Akimasa Morita, Katsumi Tanaka, Akinori Sakai, Kunitaka Menuki, Yoshiaki Yamanaka, Shiro Urata, Takeshi Oguchi, Toshikazu Kurahashi, Norimasa Iwasaki, Yuichiro Matsui, Hiroyasu Ikegami, Hiroaki Sakano, Tetsu Katsumura, Masao Nishiwaki, Toshikazu Tanaka, Yuichi Hirase, Yuri Kanno, Hiroyuki Kato, Masanori Hayashi, Shohei Omokawa, Hideo Hasegawa, Hiroyuki Gotani, Yoshitaka Tanaka, Toru Sunagawa, Rikuo Shinomiya, Rhoji Kajiwara, Etsuhiro Nakao, Takanobu Nishizuka, Yasunori Hattori, Takaaki Shinohara, Kentaro Watanabe, Nobuyuki Okui, Hiroshi Koshima, Tsuyoshi Tajika, Hiroyuki Ohi, Yoshio Kaji, Eiichi Nagayoshi, Ataru Igarashi

**Affiliations:** 1 Department of Hand Surgery, Nagoya University, 65 Tsurumai-cho, Showa-ku, Nagoya, 466-8550 Japan; 2 Department of Clinical Epidemiology and Health Economics, Tokyo University, Tokyo, Japan; 3 Department of Orthopaedic Surgery, Kindai University, Osaka Sayama, Japan; 4 Department of Hand Surgery, Niigata Hand Surgery Foundation, Seiro, Japan; 5 Department of Orthopaedic Surgery, Suzuka Kaisei Hospital, Suzuka, Japan; 6 Department of Plastic and Reconstructive Surgery, Nagasaki University, Nagasaki, Japan; 7 Department of Orthopaedic Surgery, the University of Occupational and Environmental Health, Kitakyusyu, Japan; 8 Department of Orthopaedic Surgery, Anjo Kosei Hospital, Anjo, Japan; 9 Department of Orthopaedic Surgery, Hokkaido University, Sapporo, Japan; 10 Department of Orthopaedic Surgery, Toho University Medical Center Ohashi Hospital, Tokyo, Japan; 11 Department of Orthopaedic and Hand Surgery Center, Hiratsuka Kyosai Hospital, Hiratsuka, Japan; 12 Department of Orthopaedic Surgery, Kawasaki Municipal Hospital, Kawasaki, Japan; 13 Department of Orthopaedic Surgery, Kikkoman General Hospital, Noda, Japan; 14 Department of Hand Surgery, Yotsuya Medical Cube, Tokyo, Japan; 15 Department of Orthopaedic Surgery, Shinsyu University, Matsumoto, Japan; 16 Department of Orthopaedic Surgery, Nara Medical University, Kashihara, Japan; 17 Department of Orthopaedic Surgery, Osaka Ekisaikai Hospital, Osaka, Japan; 18 Department of Orthopaedic Surgery, Hiroshima University, Hiroshima, Japan; 19 Department of Orthopaedic Surgery, Matsuyama Red Cross Hospital, Matsuyama, Japan; 20 Department of Orthopaedic and Hand Surgery Center, Chunichi Hospital, Nagoya, Japan; 21 Department of Orthopaedic Surgery, Ogori Daiichi General Hospital, Ogori, Japan; 22 Department of Orthopaedic Surgery, Daido Hospital, Nagoya, Japan; 23 Department of Orthopaedic Surgery, Nagoya Ekisaikai Hospital, Nagoya, Japan; 24 Department of Orthopaedic Surgery, Yokkaichi Municipal Hospital, Yokkaichi, Japan; 25 Department of Orthopaedic Surgery, Kani Tono Hospital, Kani, Japan; 26 Department of Orthopaedic Surgery, Gunma University, Maebashi, Japan; 27 Department of Hand and Microsurgery Center, Seirei Hamamatsu General Hospital, Hamamatsu, Japan; 28 Department of Orthopaedic Surgery, Kagawa University, Takamatsu, Japan; 29 Department of Medical Affairs, Asahi Kasei Parma Corporation, Tokyo, Japan; 30 Unit of Public Health and Preventive Medicine, Yokohama City University School of Medicine, Yokohama, Japan

**Keywords:** Health care, Medical research, Rheumatology

## Abstract

This study compared hand function and the cost-effectiveness of treatment between collagenase *Clostridium histolyticum* (CCH) injection and limited fasciectomy for patients with Dupuytren’s contracture (DC). The CeCORD-J study is a prospective, multicenter, non-randomized controlled, observational study of two parallel groups. Participants were DC patients with multiple affected fingers, including flexion contracture of the proximal interphalangeal (PIP) joint. The primary outcome was the Hand10 score, as a patient-reported outcome measure (PROM). We set secondary outcomes of EQ-5D-5L (QOL) score, degree of extension deficit, and direct cost. Propensity score adjustment was used to balance differences in patient characteristics between groups. Participants comprised 52 patients in the Collagenase group and 26 patients in the Surgery group. There were no significant differences in the Hand10 and QOL scores between the two groups at 26 weeks. Mean direct cost was 248,000 yen higher in the Surgery group than in the Collagenase group. Extension deficit angle of the PIP joint was significantly larger in the Collagenase group at 26 weeks. Although the Collagenase group showed dominance in cost-effectiveness, there was no significant difference between the two groups in hand function at 26 weeks.

## Introduction

Dupuytren’s contracture (DC) is a fibroproliferative disease of the palmar hand. DC causes symptomatic and progressive flexion contractures of the digits^1^. Flexion contracture often occurs in the metacarpophalangeal (MP) and/or proximal interphalangeal (PIP) joints of the ring and little fingers^2^. Flexion contracture of the digits influences daily activities and quality of life (QOL) by limiting hand function^3^. DC has been considered common in Caucasians of Scandinavian and Celtic ancestry, but reports on DC from Asian and African countries have increased recently^4,5^. Both genetic and environmental factors are considered to affect disease progression, and the prevalence of DC increases with age^6^.

Surgical procedures including fasciectomy, dermatofasciectomy, and needle fasciotomy have been standard treatment options for DC. Since Hurst et al. reported level 1 evidence for the utility of collagenase *Clostridium histolyticum* (CCH) injection compared with placebo in 2009, the United States Food and Drug Administration in 2010, the European Medicines Agency in 2011 and the Japanese Pharmaceuticals and Medical Devices Agency in 2015 have approved CCH injection for patients with DC^5,7^.

Hand function and degree of flexion contracture after treatment are the most important issues for patients with DC. Some reports have described upper limb function using patient-reported outcome measures (PROMs) such as Disabilities of the Arm, Shoulder and Hand (DASH) and the Michigan Hand Outcomes Questionnaire (MHQ) before and after surgical treatment for DC^8–10^. However, few papers have reported results from PROMs before and after CCH injection for patients with DC. Furthermore, very few studies have prospectively compared results between surgery and CCH injection using PROMs.

Outcomes after treatment will differ depending on which joints are affected. Generally, flexion contracture of MP joints shows better prognosis than that of PIP joints. Treatment is more difficult for PIP joints than for MP joints because of the higher frequencies of recurrence and complications. A study of patients with DC-affected PIP joints is thus necessary.

The cost-effectiveness of DC treatments has been reported from European and North American countries. Atroshi et al. reported costs for collagenase injections compared with fasciectomy in the treatment of DC as a retrospective cohort study in Sweden^11^. Although the follow-up period was as short as 6 weeks, treatment of DC with one collagenase injection cost 33% less than fasciectomy, while offering equivalent efficacy of contracture reduction. Baltzer and Binhammer in Canada estimated that injectable collagenase would be feasible for treating DC affecting a single finger if it cost significantly less than the current United States pricing^12^. On the other hand, the National Health Service (NHS) in the United Kingdom simulated the costs of treatment for patients with DC affecting more than three joints, revealing limited fasciectomy as more cost effective than percutaneous needle fasciotomy or CCH injection. CCH injection showed the worst cost-effectiveness for patients with DC affecting multiple fingers^13^. However, the costs of CCH injections, surgical procedures, and hand therapy differ between countries. Most patients are interested in the prices of treatment and often ask hand surgeons about the costs. Hand surgeons thus need cost and utility data as well as results for hand function after treatment for DC to provide patients with sufficient information on functional and economic aspects to make informed decisions before treatment.

The purpose of this study was to compare hand function and direct medical cost between CCH injection and limited fasciectomy for patients with DC-affected PIP joints. Our primary aim was to compare hand function between CCH injection and fasciectomy using the Hand10 PROM^14^. Secondary outcomes were set to compare QOL using the EQ-5D-5L^15^, patient satisfaction, degree of contracture, and direct cost. We hypothesized that no significant difference in hand function or QOL would be evident between groups, but that CCH injection would prove superior to fasciectomy in terms of cost-effectiveness.

## Materials and methods

The CeCORD-J study is a prospective, multicenter, non-randomized controlled, observational study with two parallel groups. This study provides the utmost respect for the treatment choices of patients.

### Study setting

The CeCORD-J research protocol was approved by the ethics committee of the Nagoya University Hospital (2017–0506-4) and entered in the University Hospital Medical Network Clinical Trials Registry (UMIN-CTR; number UMIN000029826). The study was approved by the academic research project committee of the Japanese Society for Surgery of the Hand (JSSH) as an official project in 2017. We invited participation from all major referral centers of hand surgery in Japan with at least one hand surgery specialist. A final total of 28 hand surgery centers accepted and joined this study. Patients were recruited from those centers. Investigators had to have specialist certification in hand surgery because CCH injection can only be performed by hand surgery specialists in Japan. All participating investigators had previously performed both CCH injection and fasciectomy before initiation of the study. Hand surgeons participating in this study held three meetings before starting this study to confirm the protocol. This study followed and respected the Declaration of Helsinki and the principles of good clinical practice.

### Ethics and dissemination

Both treatment options are standard procedures for patients with DC in Japan. This study was not a randomized controlled study for intervention selection. We have the utmost respect for patient preferences in terms of treatment selection. This study was therefore not interventional but observational in nature. This policy offers substantial benefits for patient recruitment. Our major concern was bias in the number of cases in each group. One solution to this issue is propensity score adjustment after study completion.

### Information on the trial and patient approval

Written information was used to explain the study to patients. Because this study was observational in nature, patients were able to select their preferred treatment after explanation of both treatments by hand surgery specialists. Hand surgery specialists obtained informed consent for participation from each patient after the decision-making process.

### Participants

The inclusion criteria were: (1) patients with passive extension deficit in either only PIP joints or both PIP and MP joints; (2) flexion contractures in at least two fingers of one hand; (3) age > 20 years; and (4) the ability to answer questionnaires in Japanese. The exclusion criteria were: (1) recurrent contracture in the finger to be treated; (2) contraindications for CCH injection; (3) pregnancy or planning to become pregnant; (4) need for continued anticoagulation; (5) participation in another clinical study; (6) judgement that study participation would be inappropriate by the enrolling investigator.

### Baseline assessment

Baseline demographics of patients included age, sex, family history of DC, duration of symptoms, medical history, histories of smoking and drinking, involved fingers and joints, and PROMs for the hand (Hand10), EQ-5D-5L, and satisfaction (a 5-point scale).

### Interventions

Patients requesting CCH injection were included in the Collagenase group. In this group, 0.58 mg of CCH was injected into the cord of the affected finger. One day after injection, the affected finger was manipulated under local anesthesia or wrist block. Patients could receive additional CCH injections up to 3 times in total if necessary, at intervals of > 30 days.

Patients hoping to undergo fasciectomy were included in the Surgery group. In this group, aponeurectomy was performed under general anesthesia or axillary block.

Patients in both groups received hand therapy including splinting based on the judgement of the surgeon. An attending hand surgery specialist decided whether inpatient or outpatient treatment was warranted. Generally, patients in the Surgery group required inpatient treatment, and those in the Collagenase group could be treated using either outpatient or inpatient treatment.

### Study outcomes

#### Primary outcome

The primary outcome was the PROM score for the Hand10 at 26 weeks after intervention corrected by the propensity score adjustment. The Hand10 is a valid instrument for patients with upper limb disorder, even in elderly individuals, as the questionnaire includes illustrations that provide favorable effects on reproducibility^14^. The Hand10 questionnaire consists of 10 self-reported questions designed to measure upper extremity disability and symptoms. Scores for the Hand10 range from 0 to 100, with lower numbers indicating lower levels of disability.

#### Secondary outcomes

We set the following secondary outcomes: (1) EQ-5D-5L (QOL) score; (2) patient satisfaction; (3) degrees of extension deficit and flexion of the treated joint, and (4) direct medical costs after propensity score adjustment. We also investigated: (1) complications; and (2) recurrences as other outcomes. Recurrences were assessed 1 year after treatment, and all other secondary outcomes were evaluated during the 26-week treatment after interventions. We calculated the direct costs, including any due to recurrences, during the treatment period. The incremental cost/QOL score was figured to compare the cost-effectiveness of both groups using the direct cost and QOL score at 26 weeks.

Recurrence was considered to have occurred if the degree of extension deficit worsened by more than 30° after treatment. We classified complications as: grade I, minor complications without needing any unexpected surgery or anesthesia; or grade II, major complications needing unexpected surgery under anesthesia or inpatient treatment. Each outcome was compared with baseline data.

### Discontinuation criteria

Discontinuation criteria were: 1) when the patient requested to withdraw from the study; 2) when the patient was found not to satisfy eligibility criteria after registration; 3) when continuation of the study was difficult because symptoms or complications worsened; or 4) when the hand surgery specialist decided on discontinuation for any other reason. When discontinuation occurred, data collection was stopped, but data collected up to the time of discontinuation were retained for use in the study. Differences between patients who completed and discontinued the study were analyzed by age and sex.

### Power analysis

Setting values of α = 0.05 and β = 0.8, sample size “n” was calculated as n = 16s^2^/d^2^, where “s” is the standard deviation and “d” is the difference between two groups. According to previous papers, mean DASH score at 1 year postoperatively was 12.7 (standard deviation, 3.6)^8^, and mean DASH score at 1 year after CCH injection was 7 (standard deviation, 9)^16^. We did not use DASH score, but Hand10 was expected to show similar results. We therefore used “s” as 9, and “d” as 5.7 for sample size calculations. If both groups were to include the same number of patients, a sample size of 40 per group was required.

### Statistical analysis

Propensity score adjustment was used to balance differences in patient characteristics between Collagenase and Surgery groups. In a logistic regression model with treatment assignment variables (Collagenase group = 0, Surgery group = 1) as the dependent variables, the following independent variables were introduced to obtain the propensity score (= probability of undergoing surgery) for each patient: age, sex, total number of affected joints per patient, total number of affected fingers per patient, family history of DC, duration of symptoms, smoking, drinking, past or present history of diabetes mellitus, epilepsy, history of hand trauma, malignant tumor, baseline Hand10 score, baseline degree of extension defect of the primary PIP joint, degree of extension of the primary MP joint, and baseline EQ-5D-5L (QOL) score.

Outcomes were then compared between treatment groups after adjusting for propensity scores. Specifically, a generalized linear model with treatment assignment variables (Collagenase group = 0; Surgery group = 1) and propensity scores as independent variables and outcomes as dependent variables was used to determine differences in outcomes and associated 95% confidence intervals and p-values in the collagenase-controlled Surgery group.

The outcome variables examined were: Hand10 score (weeks 1, 2, 4, 8, and 26), EQ-5D-5L (QOL) score (weeks 1, 2, 4, 8, and 26), direct cost, degree of extension deficit of the PIP (weeks 4 and 26), degree of extension deficit of the MP (weeks 4 and 26), degree of PIP flexion (weeks 4 and 26), and degree of MP flexion (weeks 4 and 26).

Prevalence and severity of complications were summarized and compared between groups using the chi-square test. We also compared baseline patient demographics between groups using the unpaired t-test or chi-square test. Baseline patient demographics included age, sex, number of involved fingers and joints, degree of extension deficit, Hand10 score, EQ-5D-5L (QOL) score, and satisfaction. Hand10 scores were compared between groups using the unpaired t-test. EQ-5D-5L (QOL) score, satisfaction, and degree of extension deficit were compared between groups using the unpaired t-test. Satisfaction was compared between groups using the Mann–Whitney U test. We compared mean direct medical costs between groups. Statistical analysis was performed using STATA version 16 software.

### Data management

We used a central monitoring system in the data center. Each hand surgery center registered anonymized patient information to the data center. Hand surgery specialists noted patient data at each time point and those data were stored at each hand surgery center. Anonymized patient data were collected at the data center by mail after finishing the follow-up period. This study was monitored by an independent assessor during patient recruitment and after completion of the study.

## Results

### Participants

**Figure 1:**
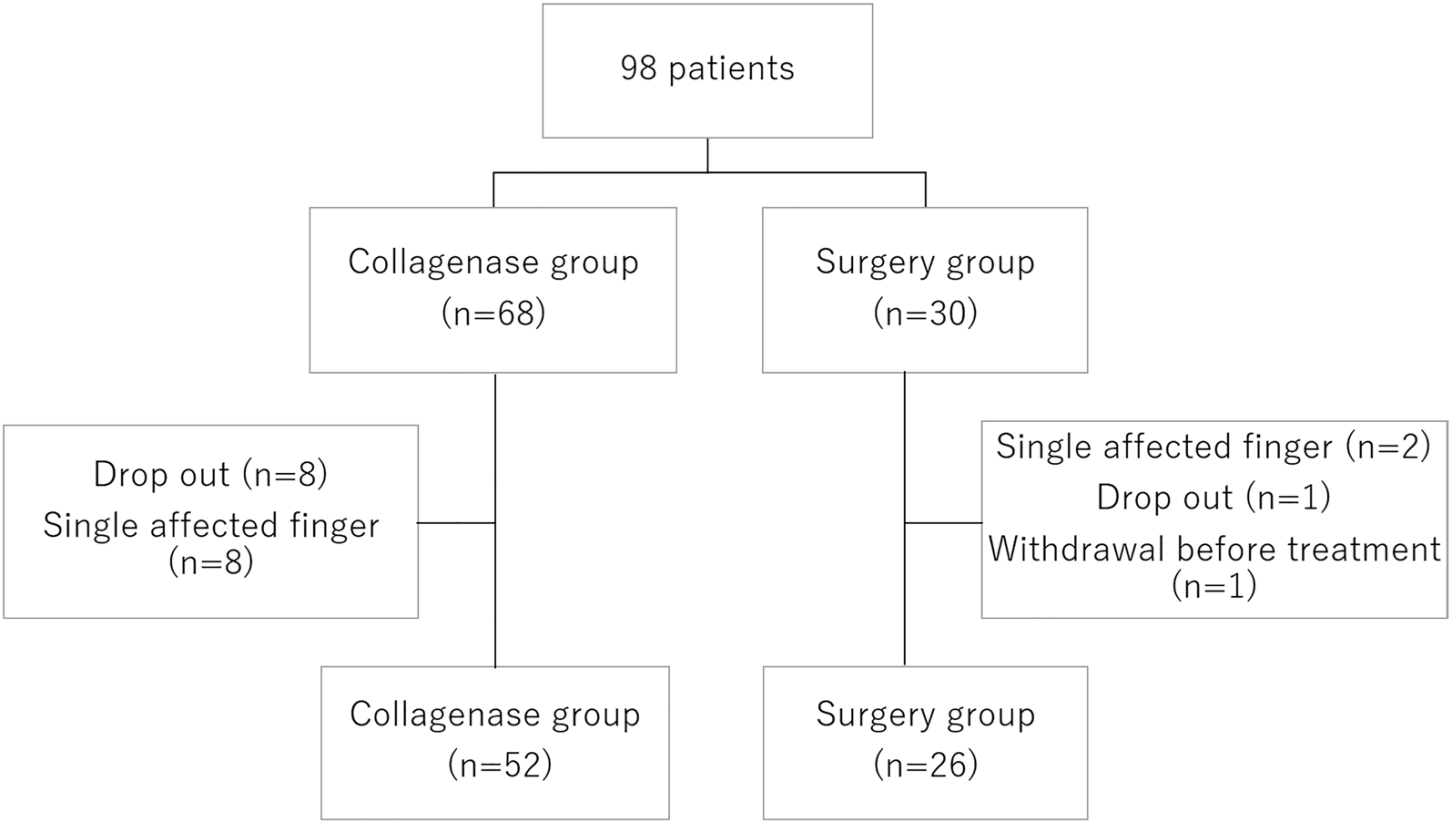
Study flowchart. Twenty patients were excluded because of single affected finger (n = 10), loss to follow-up (n = 9), or withdrawal before treatment (n = 1). n, number.

A total of 98 participants were initially enrolled in this study from April 2018 to July 2019. Of those, 20 were excluded, and data from 52 patients in the Collagenase group and 26 patients in the Surgery group (total cohort, 78 patients) were analyzed. The 20 patients were excluded due to contracture in only 1 finger (n = 10), dropout (n = 9), or voluntary withdrawal from the study before treatment (n = 1) (Fig. 1). Participant demographics are shown in Table 1. Although there was no significant difference between the two groups, the degree of extension deficit of the primary PIP joint tended to be larger in the Surgery group. In the Collagenase group, 5 of 52 patients received a second injection at different sites.

**Table 1:** Demographic and baseline clinical characteristics of participants.

Group	Collagenase	Surgery	*P* value
Number of participants	52	26	
Age, years (mean ± SD)	71 ± 8.9	70 ± 7.6	0.63
Sex	Male: 50	Male: 25	1
	Female: 2	Female: 1	
Total affected joint per patient (mean ± SD)	3.8 ± 1	4.2 ± 0.9	0.15
Total affected finger per patient (mean ± SD)	2.2 ± 0.5	2.2 ± 0.4	1
Family history of DC, n (%)	5 (10)	1 (4)	0.3
Duration of symptoms, month (mean ± SD)	14 ± 32.7	24.3 ± 35.6	0.31
**History of risk factors**			
Smoking, n (%)	15 (29)	4 (15)	0.19
Drinking, n (%)	40 (77)	21 (81)	0.78
Diabetes mellitus, n (%)	12 (23)	11 (42)	0.08
Epilepsy, n (%)	1 (2)	1 (4)	0.61
History of hand trauma, n (%)	3 (6)	1 (4)	0.71
Malignant tumor, n (%)	9 (17)	3 (12)	0.51
Hand10 score (mean ± SD)	15.5 ± 18.2	14.2 ± 15.4	0.76
Degree of extension deficit of the primary PIP joint (mean ± SD)	37.2 ± 23.6	46.9 ± 27.8	0.11
Degree of extension deficit of the primary MP joint (mean ± SD)	39.9 ± 20.5	36.5 ± 21.9	0.5

*DC* Dupuytren’s contracture, *MP* metacarpophalangeal, *PIP* proximal interphalangeal, *SD* standard deviation, *n* number.

### Primary outcome

Results from propensity score adjustment are presented in Table 2. Hand10 score was significantly higher in the Surgery group at 1 and 2 weeks, but not at other time points.

**Table 2:** Results of Hand10 score after propensity-score adjusted generalized linear modeling to compare outcomes between Collagenase and Surgery groups.

Hand10 score	Coefficient	95% Confidence interval	*P* value
1w	13.6	0.0–27.1	0.05
2w	14.3	2.0–26.6	< 0.05
4w	3.0	− 5.8–11.8	0.51
8w	2.7	− 4.8–10.2	0.48
26w	0.9	− 5.2–7.0	0.77

Coefficients (and their 95% confidence intervals) indicate the degree of each outcome in the Surgery group with reference to the Collagenase group. w, week.

### Secondary outcomes

No significant difference in EQ-5D-5L (QOL) score was seen after adjusting for propensity scores, except at 8 weeks. The direct cost was 248,000 yen (about $2275) higher in the Surgery group than in the Collagenase group on average. A significant difference in extension deficit angle of the PIP joint was apparent between groups at 26 weeks. Although flexion angle after intervention was smaller in the Surgery group, no significant difference was evident between groups after propensity score adjustment (Table 3).

**Table 3:** Results of secondary outcomes after propensity-score adjusted generalized linear modeling between Collagenase and Surgery groups.

	Coefficient	95% Confidence interval	*P* value
**EQ-5D-5L score**			
1w	− 0.030	− 0.092–0.031	0.34
2w	− 0.055	− 0.116–0.006	0.08
4w	− 0.035	− 0.100–0.029	0.28
8w	− 0.090	− 0.154 to − 0.027	< 0.01
26w	− 0.066	− 0.139–0.007	0.08
Direct cost, yen	248,375	104,249–392,501	< 0.01
**PIP degree of extension deficit**			
4w	− 12.0	− 21.6 to − 2.5	0.01
26w	− 14.5	− 25.1 to − 3.8	< 0.01
**MP degree of extension deficit**			
4w	− 5.7	− 13.8–2.4	0.17
26w	− 0.7	− 8.2–6.7	0.85
**PIP degree of flexion**			
4w	− 4.1	− 9.0–0.8	0.1
26w	− 1.5	− 5.2–2.2	0.42
**MP degree of flexion**			
4w	− 3.9	− 9.5–1.7	0.17
26w	− 3.1	− 7.1–0.9	0.13

Coefficients (and their 95% confidence intervals) indicate the degree of each outcome in the Surgery group with reference to the Collagenase group. w, week.

The outcomes of recurrence, complications, and satisfaction are summarized in Table 4. Recurrences were more frequent in the Collagenase group than in the Surgery group (18% vs 9.5%). One patient in the Collagenase group received an additional CCH injection to treat a recurrence. No one in the Surgery group underwent an additional procedure during the study period. All complications in both groups were minor without additional surgery or anesthesia. No significant difference in satisfaction was seen between groups. All patients in the Surgery group provided evaluations above neutral.

**Table 4:** Comparison of recurrence, complication, and satisfaction between Collagenase and Surgery groups.

Group	Collagenase	Surgery	*P* value
Recurrence, n (%)^a^	8 (18)	2 (9.5)	0.38
Complication, n (%)	9 (17)	2 (8)	0.25
Injection site laceration, n (%)	5 (10)		
Numbness of the finger, n (%)	1 (2)	1 (4)	
Injection site blister, n (%)	1 (2)		
Arthritis of the finger, n (%)	1 (2)		
Pneumonia, n (%)	1 (2)		
Hypertrophic scar, n (%)		1 (4)	
**Satisfaction**^**b**^			0.27
1 very satisfied	18	12	
2 satisfied	24	9	
3 neutral	4	5	
4 dissatisfied	3	0	
5 very dissatisfied	0	0	

*SD* standard deviation.

^a^The number of patients who were evaluated at 52 weeks in Collagenase and Surgery group was 45 and 21, respectively.

^b^Data of 3 patients in collagenase group was absent.

### Outcomes before propensity score adjustment

The time course for Hand10 score as primary outcome is shown in Appendix 1. Hand10 scores were significantly higher in the Surgery group than in the Collagenase group at 1 and 2 weeks after treatment.

**Figure 2:**
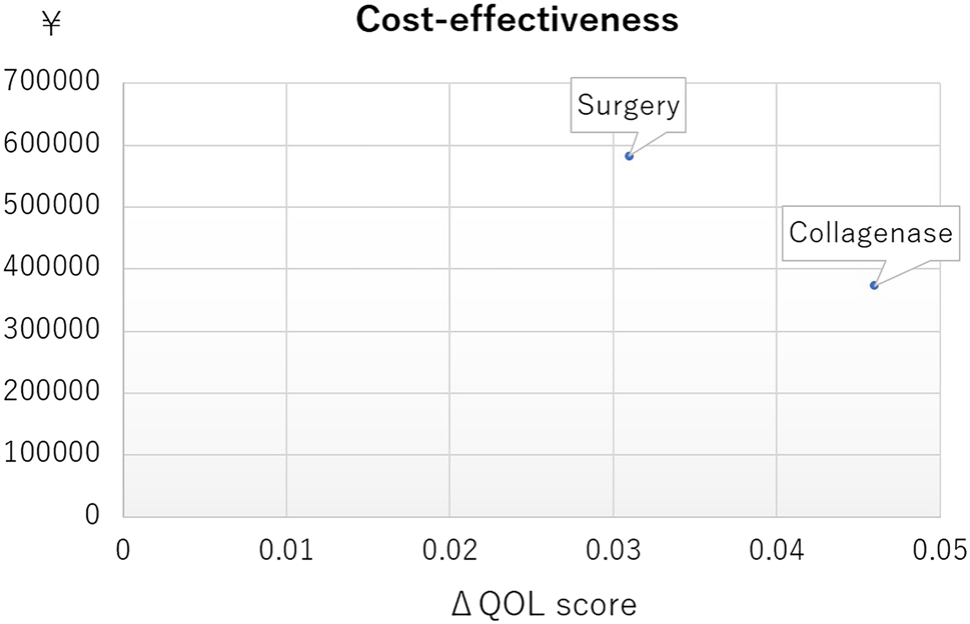
Incremental cost/QOL score at 26 weeks. The Collagenase group was dominant in cost-effectiveness at 26 weeks.

EQ-5D-5L (QOL) score was significantly higher in the Collagenase group than in the Surgery group at 2, 4, 8 and 26 weeks after treatment (Appendix 2). The direct cost for the Collagenase group was 370,000 yen (about 3395 US dollars (USD) at an exchange rate of $1 = 109 yen) on average, compared to 580,000 yen (about $5321) for the Surgery group, representing a significant difference (Table 5). The incremental cost/QOL score is shown in Fig. 2. The Collagenase group was dominant in cost-effectiveness at 26 weeks.

**Table 5:** Outcomes before propensity score adjustment.

Group	Collagenase	Surgery	*P* value
**Hand10 score**			
Baseline (mean ± SD)	15.5 ± 18.2	14.2 ± 15.4	0.76
1w (mean ± SD)	25.2 ± 23.7	46.1 ± 23.8	< 0.01
2w (mean ± SD)	18.2 ± 20.6	36.2 ± 25	< 0.01
4w (mean ± SD)	10.2 ± 13.9	16.8 ± 16.8	0.07
8w (mean ± SD)	8.1 ± 12.8	12.7 ± 14.5	0.15
26w (mean ± SD)	6.5 ± 10.3	7.9 ± 11.3	0.59
**EQ-5D-5L score**			
Baseline (mean ± SD)	0.859 ± 0.145	0.81 ± 0.121	0.14
1w (mean ± SD)	0.775 ± 0.111	0.739 ± 0.085	0.17
2w (mean ± SD)	0.816 ± 0.116	0.754 ± 0.09	< 0.05
4w (mean ± SD)	0.857 ± 0.125	0.784 ± 0.094	< 0.05
8w (mean ± SD)	0.899 ± 0.109	0.817 ± 0.117	< 0.01
26w (mean ± SD)	0.905 ± 0.134	0.841 ± 0.119	< 0.05
Direct cost, yen (mean ± SD)	372,370 ± 262,517	582,147 ± 267,204	< 0.01
**PIP degree of extension deficit**			
Baseline (mean ± SD)	37.2 ± 23.6	46.9 ± 27.8	0.11
4w (mean ± SD)	17.2 ± 19.4	12.8 ± 14.9	0.32
26w (mean ± SD)	20.6 ± 21.1	14.6 ± 15.6	0.21
**MP degree of extension deficit**			
Baseline (mean ± SD)	39.9 ± 20.5	36.5 ± 21.9	0.5
4w (mean ± SD)	7.3 ± 12.8	3.7 ± 6.4	0.18
26w (mean ± SD)	4.9 ± 9	4.5 ± 9.8	0.86
**PIP degree of flexion**			
Baseline (mean ± SD)	93.8 ± 5.3	93.7 ± 7.7	0.92
4w (mean ± SD)	91.6 ± 7.9	86.6 ± 10.4	< 0.05
26w (mean ± SD)	94.2 ± 7.1	93.5 ± 6.1	0.68
**MP degree of flexion**			
Baseline (mean ± SD)	85.9 ± 6.8	84.6 ± 6.3	0.43
4w (mean ± SD)	84.1 ± 10.9	79.1 ± 8.6	< 0.05
26w (mean ± SD)	87.5 ± 8.1	82.6 ± 6	< 0.01

*w* week, *MP* metacarpophalangeal, *PIP* proximal interphalangeal, *SD* standard deviation.

As other evaluation items, the time courses of extension deficit angle and flexion angle of the PIP and MP joints are shown in Table 5. No significant difference in extension deficit angle of the PIP or MP joint was seen between groups (Appendix 3). Flexion angles of the PIP joint at week 4 and of the MP joint at weeks 4 and 26 were significantly smaller in the Surgery group than in the Collagenase group (Appendix 4). There was no significant difference between those who completed and those who discontinued the study concerning age and sex (Appendix 5).

## Discussion

This multicenter, prospective observational study compared outcomes between CCH injection and aponeurectomy. This study was unique in that we included only patients with multiple affected fingers, including flexion contracture of the PIP joint, which is difficult to treat. This study did not randomize patients, and treatment groups were divided according to the preferences of the patient. The Collagenase group was a double of the Surgery group, because many patients preferred treatment with CCH injection.

The results of propensity score adjustment to balance differences in patient backgrounds between groups revealed significant differences in Hand10 score at 1 and 2 weeks. Patients who were injected with CCH were able to return to daily life earlier than patients who underwent surgery. However, there was no significant difference regarding hand function at 26 weeks. Zhou et al. compared CCH injection with limited fasciectomy using propensity score matching and showed significant improvements in activities of daily living, work performance, and satisfaction in their collagenase group compared to surgery group using the Michigan Hand Outcomes Questionnaire^9^. In the present study, the Collagenase group showed better PROMs than the Surgery group in the acute phase, similarly as reported by Zhou et al., however, the superiority of CCH injection in hand function disappeared at 26 weeks.

After propensity score adjustments, the EQ-5D-5L (QOL) score of the CCH injection group was significantly higher than the Surgery group at 8 weeks only. Given the difference of about 248,000 yen (about $2,275) in direct costs even if the EQ-5D-5L (QOL) score is almost equivalent between groups, collagenase appears to offer better short-term cost-effectiveness by 26 weeks, even for those patients with multiple affected joints, including PIP joints. The price of collagenase in Japan is 197,202 yen and 24,900 yen is added as a procedure fee for a total of 222,102 yen (about $2,038). On the other hand, the cost of surgery is 224,800 yen for two or three affected fingers plus 1,700 yen for regional anesthesia for a total of 226,500 yen (about $2,078) (Appendix 6). No significant difference in cost was seen between collagenase injection and surgery itself, but hospitalization and rehabilitation visits led to a difference in total direct costs.

CCH injection is feasible for treating DC affecting a single finger, according to a Canadian cost-utility analysis^12^. Conversely, the NHS has estimated that CCH injection would yield the worst cost effectiveness for DC involving more than three joint contractures^13^. Although the follow-up period was short, at 26 weeks, the present study provided evidence for the cost effectiveness of CCH injection even for hands affected in multiple fingers, including PIP joints.

No significant difference in satisfaction was seen between groups using the 5-point scale, and all patients in the Surgery group provided evaluations above neutral. The satisfaction of patients in both groups was not poor, regardless of cost-effectiveness.

The number of complications tended to be higher in the Collagenase group. Skin laceration was the most frequent complication in the Collagenase group, and no serious complications showing a causal relationship to the treatment were encountered. Recurrence also tended to be more frequent in the Collagenase group. Recurrences were reported in 160 of 199 digits (80%) at an average of 7.2 years after collagenase injection despite a heterogeneous patient population^17^. Furthermore, Yoon et al. simulated recurrent DC in a 60-year-old patient and concluded that collagenase injections are not a cost-effective intervention and should not be preferred over percutaneous needle aponeurotomy or limited fasciectomy^18^. Leafblad et al. retrospectively reviewed 848 interventions for DC, and reported 2-year reintervention rates following needle aponeurotomy, collagenase, and fasciectomy of 24%, 41%, and 4%, respectively, and 5-year rates of 61%, 55%, and 4%, respectively^19^. Cumulative costs for possible reinterventions thus also need to be taken into consideration.

Flexion angles of both the PIP and MP joints after treatment tended to be reduced in the Surgery group. This phenomenon often occurs clinically, and although finger extension improves after surgery, bending the joint deeply when grasping becomes difficult. This effect is attributed to swelling of the fingers, surgical scarring, and tendon adhesion. Rehabilitation measures such as passive range-of-motion training were required after surgery. In the Collagenase group, the decrease in flexion angle was slight and rehabilitation was often not required, which was considered to affect the cost.

This study provided comprehensive evidence for a comparison of collagenase injection and surgery for DC with contractures of more than two fingers including a PIP joint. However, some limitations must be kept in mind when interpreting the findings from this study. First, some degree of selection bias would be present, because this was a prospective observational study and patients were not randomly assigned. Surprisingly, the two groups showed no significant differences in background characteristics other than the number of patients. Furthermore, propensity score adjustments were performed to balance differences in patient background between groups without reducing the number of participants eligible for analysis.

Second, there was a very low patient to care provider ratio. As the proportion of patients with Dupuytren's contracture in Japan is smaller than in either Europe or the United States, specialists from 28 hand surgery centers worked in collaboration to ensure that the study recruited an adequate number of participants. To reduce confounding as much as possible, this study was conducted only by hand surgery specialists of the Japanese Society for Surgery of the Hand (JSSH) who were experienced in both administering CCH injections and performing surgeries for Dupuytren’s contracture.

Third, recurrence was evaluated at 52 weeks, while all other outcomes had a follow-up period of 26 weeks. Although the financial issue of multi-institutional, long-term follow-up study needs to be considered, comparison of longer-term results is certainly desirable. However, for the elderly individuals who represent the majority of patients with this disease, short-term results are also important.

Finally, finger joint angles were measured by either the hand surgeon or a hand therapist without blinding. At the same time, this study was monitored by an independent assessor during patient recruitment and after completion of the study following the principles of good clinical practice.

## Conclusion

For DC with multiple affected fingers, including flexion contracture of the PIP joint, this multicenter, prospective, observational study was conducted by dividing patients into an injectable Collagenase group and a Surgical group, giving priority to the wishes of the patient. Propensity scores were adjusted to balance differences in patient background between groups. The Hand10 scores, our primary outcome measure, showed no significant differences between the two groups at 26 weeks, nor did the EQ-5D-5L (QOL) scores. The direct cost was 248,000 yen (about 2,275 US dollars at the exchange rate of $1 = 109 yen) higher in the Surgery group than in the Collagenase group. Short-term cost-effectiveness appeared better in the Collagenase group. However, the Collagenase group had both significantly greater degrees of extension deficit of the PIP joint and more frequent minor complications and recurrences after treatment.

The CeCORD-J study was able to compare comprehensive data on hand function and cost-effectiveness between collagenase injection and surgery. The results of this study will help in treatment decisions.

## Supplementary Information

Supplementary Information 1.

Supplementary Information 2.

Supplementary Information 3.

Supplementary Information 4.

Supplementary Information 5.

Supplementary Information 6.

Supplementary Legends.
