# Supplementary figures and images for: The CeCORD-J study on collagenase injection versus aponeurectomy for Dupuytren's contracture compared by hand function and cost effectiveness
Source: Sci Rep. 2022 May 31;12:9094. doi: 10.1038/s41598-022-12966-z (PMC9156707; doi:10.1038/s41598-022-12966-z)

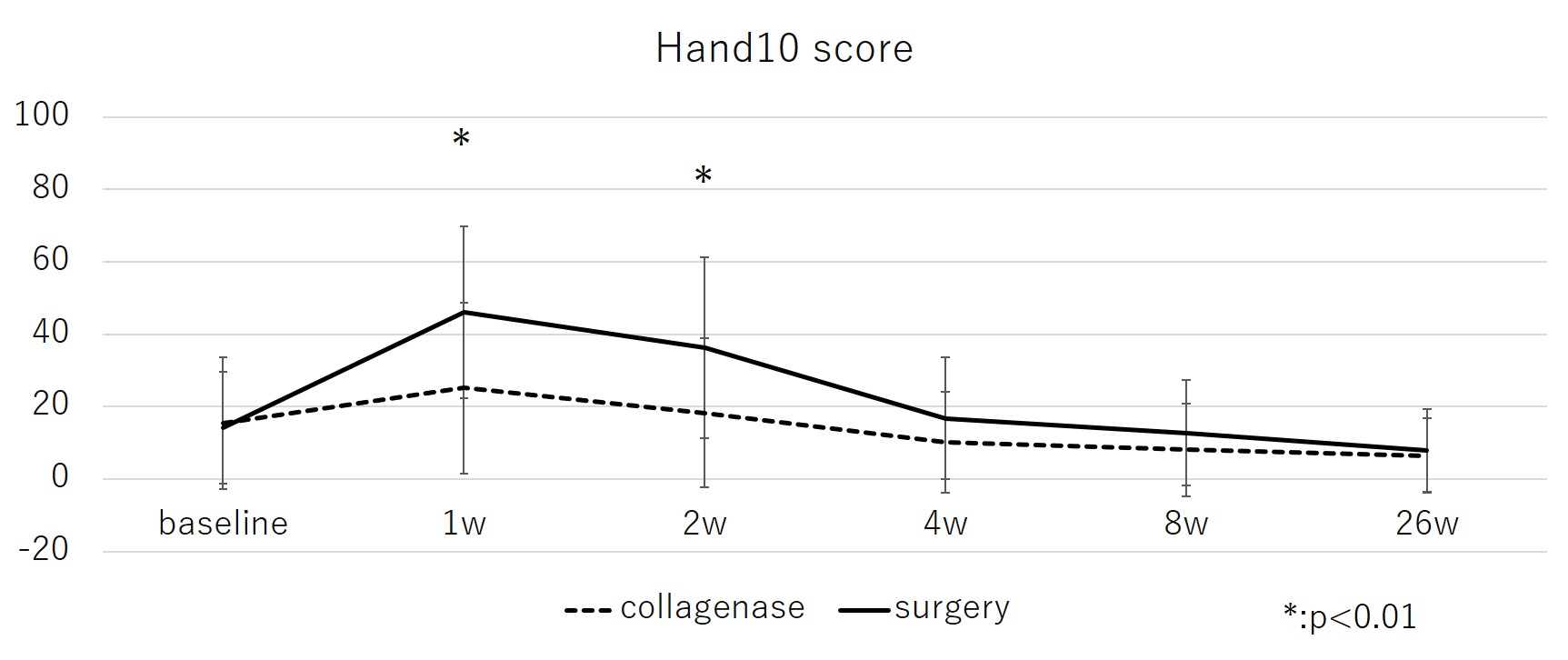

Supplement: Supplementary file 1 — Supplementary Information 1. [file 41598_2022_12966_MOESM1_ESM.jpg]

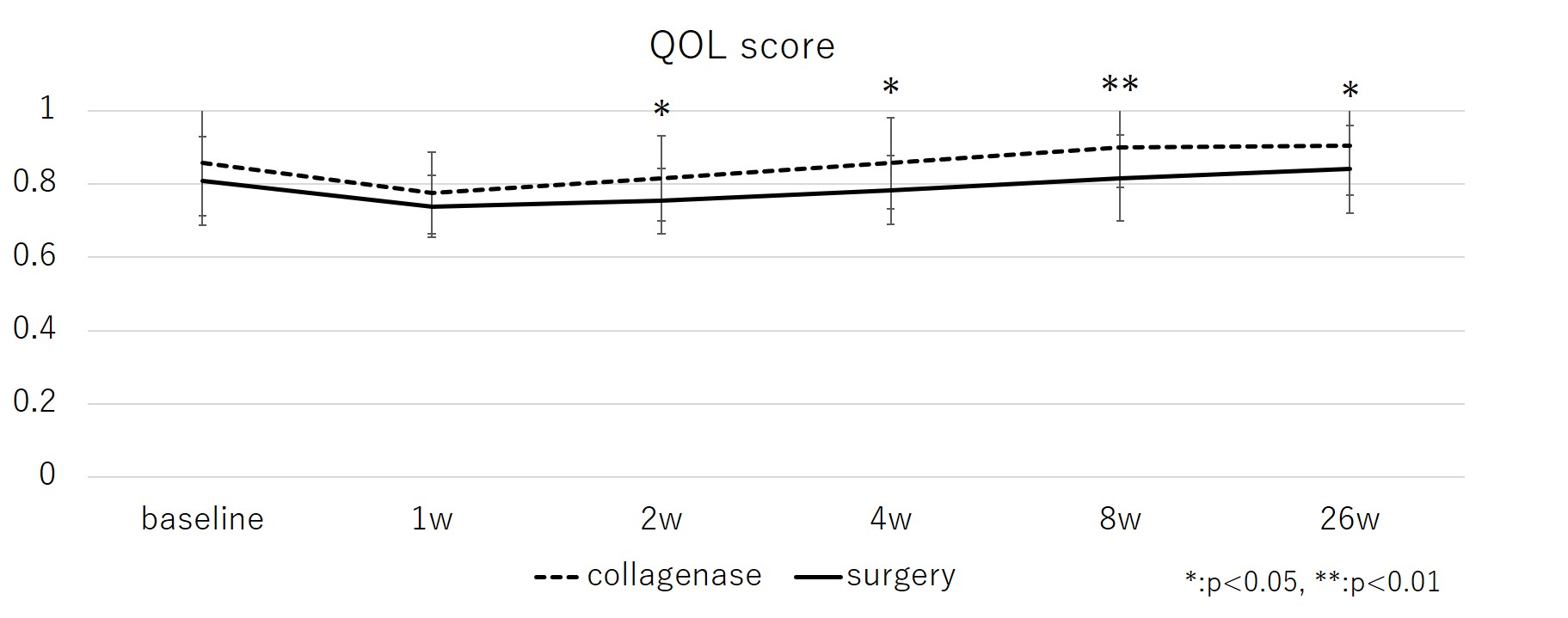

Supplement: Supplementary file 2 — Supplementary Information 2. [file 41598_2022_12966_MOESM2_ESM.jpg]

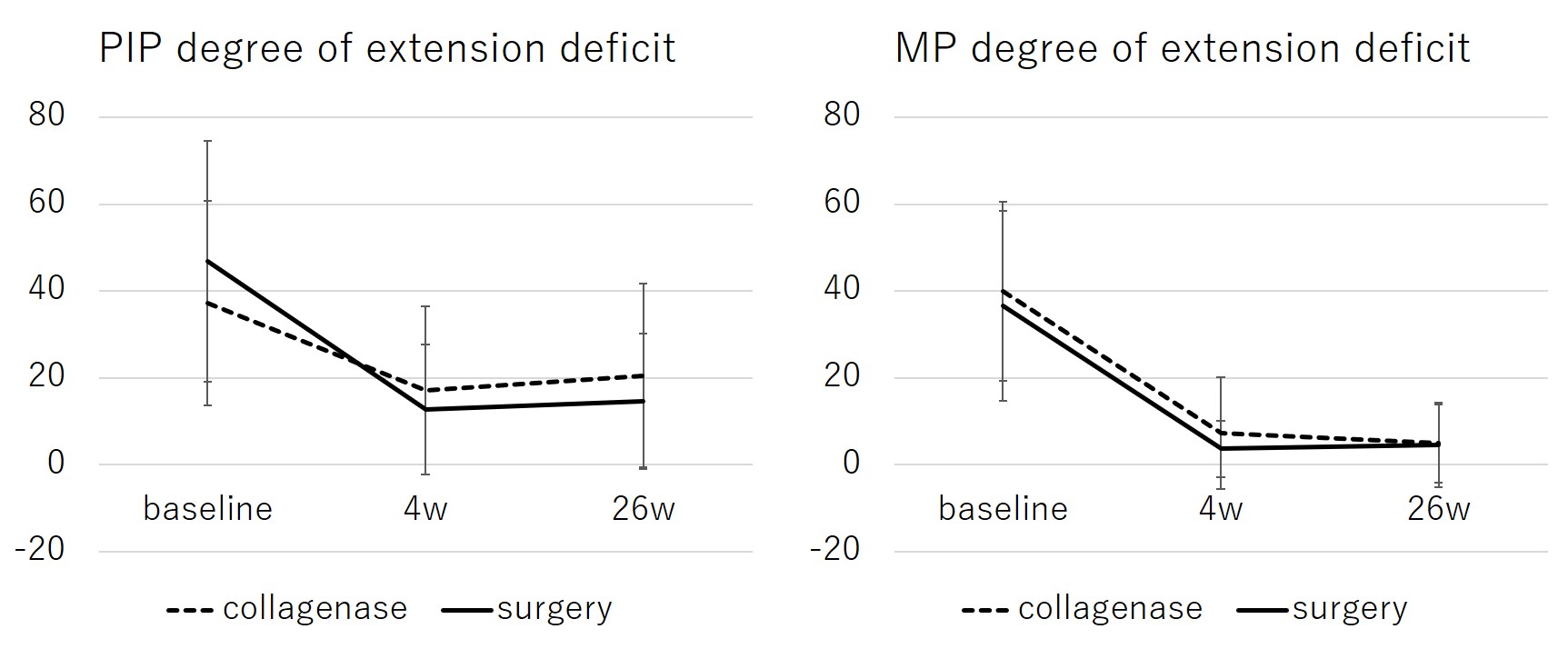

Supplement: Supplementary file 3 — Supplementary Information 3. [file 41598_2022_12966_MOESM3_ESM.jpg]

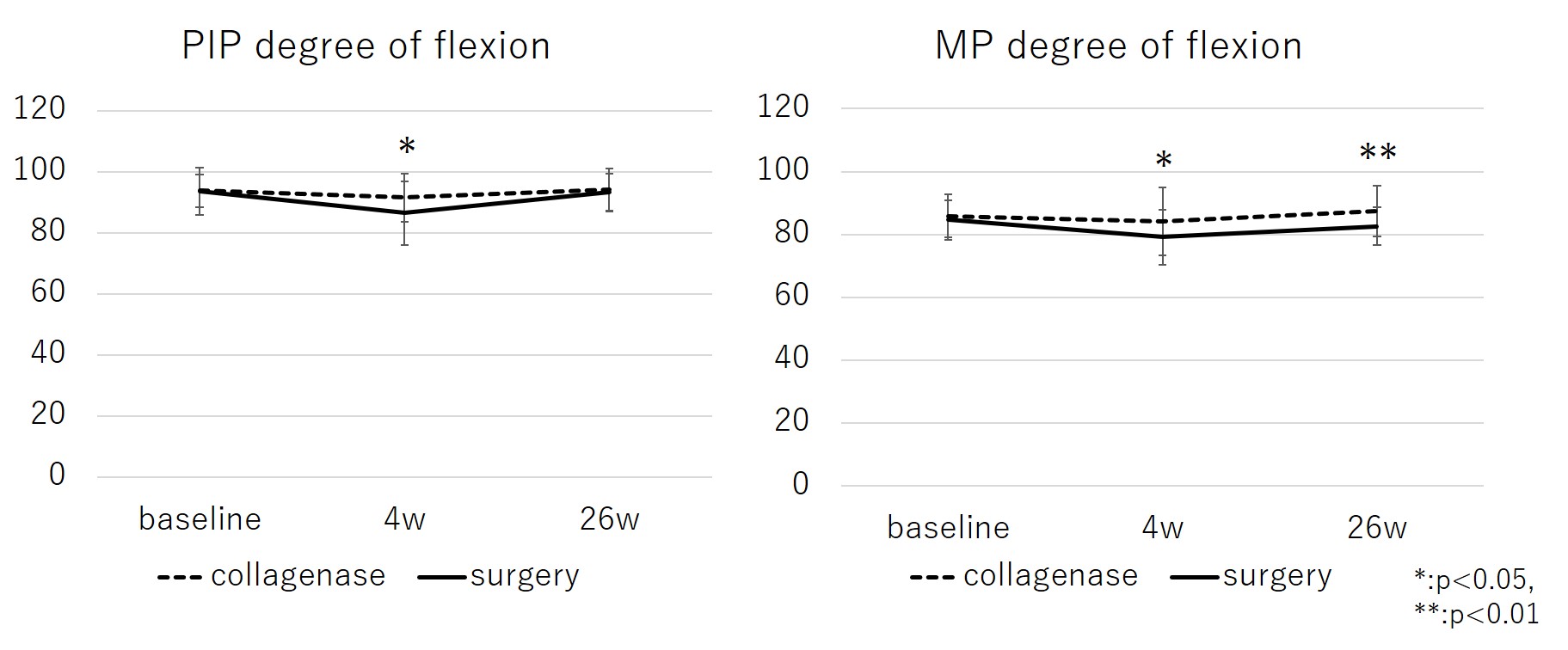

Supplement: Supplementary file 4 — Supplementary Information 4. [file 41598_2022_12966_MOESM4_ESM.jpg]
