# Supplementary material for: The CeCORD-J study on collagenase injection versus aponeurectomy for Dupuytren's contracture compared by hand function and cost effectiveness
Source: Sci Rep. 2022 May 31;12:9094. doi: 10.1038/s41598-022-12966-z (PMC9156707; doi:10.1038/s41598-022-12966-z)
Supplement: Supplementary file 5 — Supplementary Information 5. [file 41598_2022_12966_MOESM5_ESM.docx]

**Appendix 5 Comparison of patients who completed and discontinued the study**

|  | **Completed** | **Discontinued** | **P value** |
| --- | --- | --- | --- |
| **Number of patients** | 78 | 20 |  |
| **Age, years (mean ± SD)** | 70.8±8.5 | 72.9±9.7 | 0.34 |
| **Sex** | Male: 75 | Male: 19 | 0.82 |
|  | Female: 3 | Female: 1 |  |

“Completed” means patients who participated entire study.

“Discontinued” means patients who met the discontinuation criteria.
