# Supplementary material for: The CeCORD-J study on collagenase injection versus aponeurectomy for Dupuytren's contracture compared by hand function and cost effectiveness
Source: Sci Rep. 2022 May 31;12:9094. doi: 10.1038/s41598-022-12966-z (PMC9156707; doi:10.1038/s41598-022-12966-z)
Supplement: Supplementary file 6 — Supplementary Information 6. [file 41598_2022_12966_MOESM6_ESM.docx]

**Appendix 6. Cost of the series of procedures in both groups**

|  | **Collagenase** | **Surgery** |
| --- | --- | --- |
| **Price, yen** | 197,202 |  |
| **Treatment fee, yen** | 24,900 | 224,800 |
| **Regional anesthesia fee, yen** |  | 1,700 |
| **Total cost, yen** | 222,102 | 226,500 |
