# Supplementary material for: The CeCORD-J study on collagenase injection versus aponeurectomy for Dupuytren's contracture compared by hand function and cost effectiveness
Source: Sci Rep. 2022 May 31;12:9094. doi: 10.1038/s41598-022-12966-z (PMC9156707; doi:10.1038/s41598-022-12966-z)
Supplement: Supplementary file 7 — Supplementary Legends. [file 41598_2022_12966_MOESM7_ESM.docx]

**Appendix 1. Hand10 scores without propensity score adjustment**

Hand10 score was assessed as the primary outcome. Hand10 scores were significantly higher in the Collagenase group compared to the Surgery group at both 1 week and 2 weeks. w, week

**Appendix 2. EQ-5D-5L (QOL) scores without propensity score adjustment**

QOL scores were significantly higher in the Collagenase group than in the Surgery group from 2 weeks to 26 weeks. w, week

**Appendix 3. Degree of extension deficit without propensity score adjustment**

No significant difference was identified between groups. w, week

**Appendix 4. Degree of flexion angle without propensity score adjustment**

Mean flexion angle of the PIP joint was significantly smaller in the Surgery group than in the Collagenase group at 4 weeks. Mean flexion angle of the MP joint was significantly smaller in the Surgery group than in the Collagenase group at both 4 and 26 weeks. w, week

**Appendix 5. Comparison between patients completing and discontinuing study**

**Appendix 6. Cost of the series of procedures in both groups**
